# Comparative genomics and time-course transcriptomics uncover homoeologous exchange events reshaping glucosinolate metabolism in *Brassica napus*

**DOI:** 10.1186/s43897-026-00241-y

**Published:** 2026-08-04

**Authors:** Yizhou He, Zetao Bai, Zengfeng Wang, Yu-Xuan Bai, Qinlin Ke, Fan Liu, Chaobo Tong, Aixia Gu, Marta Francisco, Deyang Xu, Lijiang Liu, Qing-Yong Yang, Shengyi Liu, Wen-Hui Lin, Yuanyuan Zhang

**Affiliations:** 1 The Key Laboratory of Biology and Genetic Improvement of Oil Crops, The Ministry of Agriculture and Rural Affairs of PRC, Oil Crops Research Institute, Chinese Academy of Agricultural Sciences, Wuhan, Hubei 430062 China; 2 National Key Laboratory of Crop Genetic Improvement, Hubei Hongshan Laboratory, Huazhong Agricultural University, Wuhan, Hubei 430070 China; 3 College of Horticulture, State Key Laboratory of North China Crop Improvement and Regulation, Key Laboratory of Vegetable Germplasm Innovation and Utilization of Hebei, Hebei Agricultural University, Baoding, Hebei 071001 China; 4 State Key Laboratory of Microbial Metabolism, Joint International Research Laboratory of Metabolic and Developmental Sciences, School of Life Sciences and Biotechnology, Shanghai Jiao Tong University, Shanghai, 200240 China; 5 Group of Genetics, Breeding and Biochemistry of Brassicas, Misión Biológica de Galicia, Pontevedra, 36080 Spain; 6 DynaMo Center, Department of Plant and Environmental Sciences, Faculty of Science, University of Copenhagen, Frederiksberg, Denmark; 7 Key Laboratory of Western Resources and Modern Biotechnology of the Ministry of Education, Key Laboratory of Biotechnology of Shaanxi Province, College of Life Sciences, Northwest University, Xi’an, Shaanxi 710069 China

**Keywords:** *Brassica napus*, Structural variation, Homoeologous exchange, Glucosinolate metabolism, Time-course transcriptome, MYB28 transcription factor

## Abstract

Improving glucosinolate (GSL) profiles in rapeseed (*Brassica napus*)—high in leaves for pathogen resistance but low in seeds for meal quality—is a key breeding goal, yet its genetic basis remains unclear. Here, we present a chromosome-level genome assembly for ZY821, an elite high-GSL variety, generated using long-read sequencing and Hi-C scaffolding. Comparative analysis with the low-GSL variety ZS11 identified three major homoeologous exchange (HE) events and extensive structural variation. Notably, an A09–C09 HE event replaced the low-expression *BnaC09.MYB28* allele with the high-expression *BnaA09.MYB28* allele, resulting in elevated *MYB28s* expression and thereby increased GSL accumulation in ZY821, whereas a deletion of *BnaA09.MYB28* in ZS11 significantly reduced the expression of multiple putative downstream targets in the GSL biosynthesis pathway, leading to a reduction in GSL content. This mechanism was supported by population-level HE analysis and time-course transcriptomes across 116 RNA-Seq samples. Furthermore, joint differential expression and co-expression network analyses uncovered several novel candidate genes implicated in GSL metabolism. Collectively, our study provides new mechanistic insights into the genetic control of GSL accumulation, with significant implications for breeding optimized GSL profiles.

**Supplementary Information:**

The online version contains supplementary material available at 10.1186/s43897-026-00241-y.

## Core

A high-quality genome assembly of high-GSL rapeseed ZY821 reveals structural variations, including an A09-C09 homoeologous exchange and *BnaA09.MYB28* deletion, that underpin natural glucosinolate variation. Multi-tissue time-course transcriptomics confirms *BnaA09.MYB28* as a master regulator controlling downstream glucosinolate biosynthesis genes, providing insights for breeding varieties with optimized glucosinolate distribution.

## Gene & accession numbers

*BnaZ8A090007320 (BnaA09.MYB28), BnaZ8A090007140 (BnaA09.MYB34), BnaZ8C050054580 (BnaC05.BCAT4), BnaZ8A030039380 (BnaA03.BCAT4), BnaZ8A060012150 (BnaA06.CYP79F1), BnaZ8A040009450 (BnaA04.CYP83A1), BnaZ8C040050800 (BnaC04.CYP83A1), BnaZ8A070041410 (BnaA07.SOT18), BnaZ8A090019150 (BnaA09.SOT18), BnaZ8A020048390 (BnaA02.MAM1), BnaZ8A040009450 (BnaA04.CYP83A1), BnaZ8C040050800 (BnaC04.CYP83A1), BnaZ8A030021240 (BnaA03.LBD15), BnaZ8A040032580 (BnaA04.LBD15), BnaZ8C040079720 (BnaC04.LBD15), BnaZ8A010013800 (BnaA01.MYB85), BnaZ8A040035110 (BnaA04.WRKY12), BnaZ8C040082690 (BnaC04.WRKY12), BnaZ8C040050910* (ERF/AP2 family),* BnaZ8C070071540 (BnaC07.VAL2), BnaZ8C080064360 (BnaC08.IAA5), BnaZ8A020013640* (aspartate kinase 3), *BnaZ8A030028630* (methionine sulfoxide reductase B3), *BnaZ8C0900536670* (sulfotransferase 12), and *BnaZ8C090075400* (sulfate transporter 2;1) were analyzed in this study. The genome assembly sequence, gene annotations, SV information and RNAseq data are available at the BnaOmics Portal (https://BnaOmics.ocri-genomics.net).

## Introduction

Glucosinolates (GSLs) are a large group of sulfur-rich, amino acid-derived secondary metabolites prevalent in plants of the order Brassicales (Qin et al. 2023; Seo and Kim 2017). Due to their diverse biological properties, GSLs have garnered significant research interest in recent decades. On one hand, GSLs and their hydrolysis products function as defense compounds, playing important roles in protecting plants against microbial pathogens and herbivorous insects, thereby empowering plants with adaptability to challenging environments (Ahmad et al. 2024; Bell et al. 2022; Clay et al. 2009; Ding et al. 2021). On the other hand, rapeseed meal serves as a valuable and cost-effective protein source for livestock, yet certain GSLs, such as the aliphatic GSL, progoitrin (2-hydroxy-3-butenyl GSL), accumulate abundantly in seeds and can diminish the nutritional value of seed-based, protein-rich animal fodder. This occurs through negative effects on palatability, feed intake, nutrient utilization, and even thyroid function in animals (Nour-Eldin et al. 2017; Tayo et al. 2012). Therefore, elucidating the genetic basis of GSL biosynthesis and accumulation in cruciferous crops is essential to reconcile the apparent conflict between the environmental fitness benefits of GSLs for plants and their economic value for human and animal use.

Structurally, GSLs consist of a sulfonated oxime group linked to a thioglucose group, and an amino acid-derived R group (side chain). Based on the precursor amino acid of the side chain, GSLs are classified into three categories: aliphatic GSLs derived from Met, Ala, Leu, Ile, or Val; benzenic GSLs from Phe or Tyr; and indolic GSLs from Trp (Halkier and Gershenzon 2006). In the model plant *Arabidopsis thaliana*, the GSL biosynthesis and metabolic pathways are well characterized (Sønderby et al. 2010b). The pathway initiates from the respective amino acid and proceeds through three main stages (Sønderby et al. 2010b). First, the carbon chain of non-tryptophan amino acids undergoes one to six elongation cycles, producing chain-elongated derivatives (Mitreiter and Gigolashvili 2021; Sønderby et al. 2010b). Second, the core GSL structure is formed. Third, the amino acid side chains can undergo various secondary modifications, including hydroxylation, oxidation, methoxylation, desaturation, or benzoylation (Blažević et al. 2020; Halkier and Gershenzon 2006; Kliebenstein 2001; Mitreiter and Gigolashvili 2021; Sønderby et al. 2010b). Nearly all enzymatic steps in *Arabidopsis* GSL biosynthesis have been identified (Bell 2019; Harun et al. 2020). For instance, key enzymes in side chain elongation include *BRANCHED-CHAIN AMINOTRANSFERASE4* (*BCAT4*), *BCAT6*, *BCAT3, isopropylmalate dehydratase1* (*IPMI1*)*, IPMI2, 3-isopropylmalate dehydrogenase* 1 (IPMDH1), and the partially redundant *Methylthioalkyl Malate Synthase*s *MAM1*, *MAM2*, and *MAM3* (He et al. 2009; Knill et al. 2009; Kroymann et al. 2001; Lächler et al. 2015; Schuster et al. 2006; Textor et al. 2007). Enzymes involved in core structure formation include *Cytochrome P450 79F1* (*CYP79F1*), *CYP79F2*, *CYP83A1, CYP83B1, CYP79B2*, *CYP79B3*, *GLUTATHIONE S-TRANSFERASE F11* (*GSTF11*), and sulfotransferases *SOT16–18* (Bak et al. 2001; Chen et al. 2003; Hemm et al. 2003; Klein and Papenbrock 2009; Mikkelsen et al. 2000; Reintanz et al. 2001; Zhang et al. 2022; Zhao et al. 2002). Secondary modifications are significantly influenced by flavin-containing monooxygenases *FMO*_*GS-OXs*_ and *2-oxoglutarate-dependent dioxygenases AOP2–3* (Hansen et al. 2007; Kliebenstein 2001; Kliebenstein et al. 2001; Li et al. 2008). Furthermore, three R2R3-MYB transcription factors (TFs) – MYB28, MYB29, and MYB76 – are central regulators of aliphatic GSL synthesis, while MYB34, MYB51, and MYB122 regulate indolic GSL synthesis (Hirai et al. 2007; Sønderby et al. 2010a; Frerigmann and Gigolashvili 2014). Notably, MYB28 acts as the predominant positive regulator of numerous critical genes in the aliphatic GSL pathway, including *MAM1*, *MAM3*, *BCAT4*, *CYP79F1*, *CYP79F2*, *CYP83A1*, *SOT17*, *SOT18*, and *AOP2* (Sønderby et al. 2007; Hirai et al. 2007; Gigolashvili et al. 2008). GSLs are primarily synthesized in source tissues like leaves, silique valves, and funiculi. Their transport into seeds depends on plasma membrane-localized exporters (UMAMIT29, UMAMIT30, UMAMIT31) and importers (GTR1, GTR2, GTR3) (Nour-Eldin and Halkier 2008; Nour-Eldin et al. 2012; Xu et al. 2023).

Allotetraploid *Brassica napus* (2*n* = 4*x* = 38, AACC) is a globally important crop for edible oil and animal feed. However, high seed GSL content (SGC) reduces oil quality and poses health risks for animals consuming the residual cake meal (Walker and Booth 2015; Nour-Eldin et al. 2017). To reduce SGC in rapeseed, "double-low" breeding programs (low seed GSL and low seed erucic acid) were implemented from 1970s (Hasan et al. 2008). Today, most modern oilseed rape varieties are "double-low quality", possessing zero erucic acid and low SGC. During this process, the Polish spring rapeseed variety Bronowski, identified in 1969 as a low-GSL source, became the primary donor of the low GSL character to modern varieties, creating a narrow genetic bottleneck (Hasan et al. 2008; Kondra and Stefansson 1970; Tu et al. 2025). Reduced SGC is often accompanied by lower leaf GSL levels, rendering modern varieties more susceptible to pests, birds, and pathogens (Mithen 1992). Breeders worldwide seek germplasm combining high leaf GSL for enhanced pest/disease resistance and low seed GSL for improved oil and feed quality. However, to our knowledge, no such variety with optimal levels of both traits has been identified. Therefore, dissecting the genetic basis of GSL biosynthesis and accumulation in green tissues versus seeds is crucial for optimizing GSL distribution in future breeding. High-quality genome assemblies of representative rapeseed varieties like Darmor-*bzh*, ZS11, and Xiang5A provide valuable resources (Chalhoub et al. 2014; Chen et al. 2021; Li et al. 2023; Rousseau-Gueutin et al. 2020; Song et al. 2020). However, none of these studies involved high seed GSL varieties, hindering the precise characterization of GSL-related genes and the genomic basis of GSL differentiation between high and low GSL varieties in polyploid rapeseed.

In the present study, we generated a high-quality de novo genome assembly for Zhongyou 821 (ZY821), an elite semi-winter variety widely grown from the 1980s to 1990s known for its high GSL content. This assembly facilitates detailed gene synteny analysis, structural variant (SV) identification, and the cloning of GSL-related genes. Furthermore, comparative analysis of multi-tissue time-course transcriptomic data from ZY821 and the low-GSL representative Zhongshuang 11 (ZS11), aligned to this new reference sequence, enabled to elucidate the differentiation in GSL biosynthesis and translocation between these varieties. This study provides deeper insights into the genetic basis of the rapeseed GSL trait.

## Results

### High-quality genome assembly of a high-GSL representative variety ZY821

**Fig. 1:**
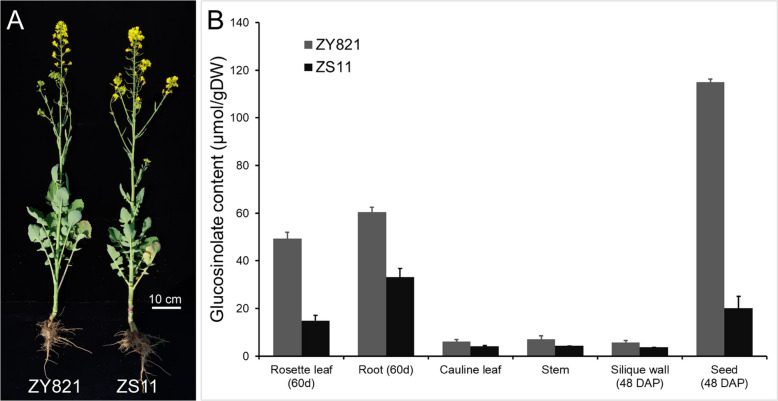
The phenotypes of high Glucosinolate variety ZY821 and low Glucosinolate variety ZS11. **A** The phenotypes at flowering time stage. d, days after sowing; DAP, days after pollination. **B** Glucosinolate content at different tissues. μmol/gDW, μmol GSL/g seed dry weight. Data are presented as means and were quantified with four biological replicates (*n* = 4)

To address the lack of high-quality genomic resources for high-GSL varieties in high GSL trait research, we selected ZY821, a semi-winter *B. napus* variety with high GSL content in both vegetative and reproductive organs (Fig. 1), for genome assembly. The ZY821 genome was sequenced using PacBio HiFi and Illumina technologies. Approximately 99.92 Gb of Illumina paired-end short reads were generated for initial genome size estimation via K-mer analysis, yielding an estimate of ~ 1,201.22 Mb. Subsequently, PacBio HiFi generated 30.35 Gb of data (~ 25.27-fold coverage, N50 = 18.89 kb), which was assembled into a 1,040.7 Mb draft genome using Hifiasm (Cheng et al. 2021).

To enhance the assembly to chromosome-level, we generated 90.37 Gb (~ 75.23-fold coverage) of Hi-C sequencing data. The final assembled ZY821 genome size was approximately 1,013.61 Mb (contig N50 = 14.23 Mb), with 978.49 Mb (96.53%) successfully anchored onto 19 pseudochromosomes and a longest contig of 51.28 Mb, indicating high assembly quality (Table 1). Using the quartet toolkit (Lin et al. 2023) with default parameters, we identified 20 telomeres and marked 155 gaps within the genome (Fig. S1; Table 2; Table S1). Our assembly is longer than previous assemblies of Darmor-*bzh* v4.1, Darmor-*bzh* v10, and comparable in length to ZS11, currently the most widely used reference genome, and the gap-free assembly Xiang5A (Table 2; Table S2). Collinearity analyses further supported this, showing higher collinearity between our ZY821 pseudochromosomes and those of ZS11 and Xiang5A compared to other assemblies (Fig. S2, S3).

**Table 1:** Statistics of ZY821 genome assembly

Genome assembly	ZY821
Assembly length (bp)	1,013,613,422
Longest contig (bp)	51,278,867
Number of contigs	744
Mean contig length (bp)	1,362,286
Contig N50 (bp)	14,232,360
Merqury (QV)	33.94
Merqury (Completeness (%))	97.53
Anchor ratio	96.53%
Chromosome length (bp)	978,490,483
Protein‐coding genes	117,985
GC (%)	37.26%
LAI	10.91
LTR (%)	39.64%
Total repeative sequence	58.67%
Retroelements	30.57%
LTR elements	27.67%
LTR-Ty1/Copia	9.45%
LTR-Gypsy/DIRS1	13.37%
DNA transposons	8.58%
Gene number	117,985
Mean gene length (bp)	2,630
Mean CDS length (bp)	210
CDS number per mRNA	6.41
BUSCO	99.80%
Single-copy BUSCOs (%)	9.70%
Duplicated BUSCOs (%)	90.10%

**Table 2:** Assembly statistics of rapeseed genomes

Assembly name	ZY821	X5A	ZS11	Darmor-*bzh* v10	Darmor-*bzh* v4.1
Principal sequencing technology	HiFi Hi-C	ONT HiFi Hi-C	PacBio Hi-C	ONT	454 sequencing
Total size of the assembled sequence (Mb)	1,013	1,045	1,008	924	850
Anchored chromosome (Mb)	978	1,005	961	867	645
Sequence was anchored to chromosomes (%)	96.5	96.2	95.3	93.8	75.8
Number of contigs	744	19	8,773	505	44,837
Contig N50 (Mb)	14.2	50.7	1.51	11.47	0.04
Number of scaffolds	\	19	3,332	237	\
Gaps	155	0	5,460	268	\
Number of telomeres	20	25	0	0	0
Number of annotated genes	117,985	124,744	100,919	108,190	101,040
Average gene length (bp)	2630	2493	2061	2011	1953
Total size of TEs (Mb)	594.71	596.80	574.70	497.66	342.34
BUSCOs (%)	99.80	99.20	98.50	98.60	97.60

**Fig. 2:**
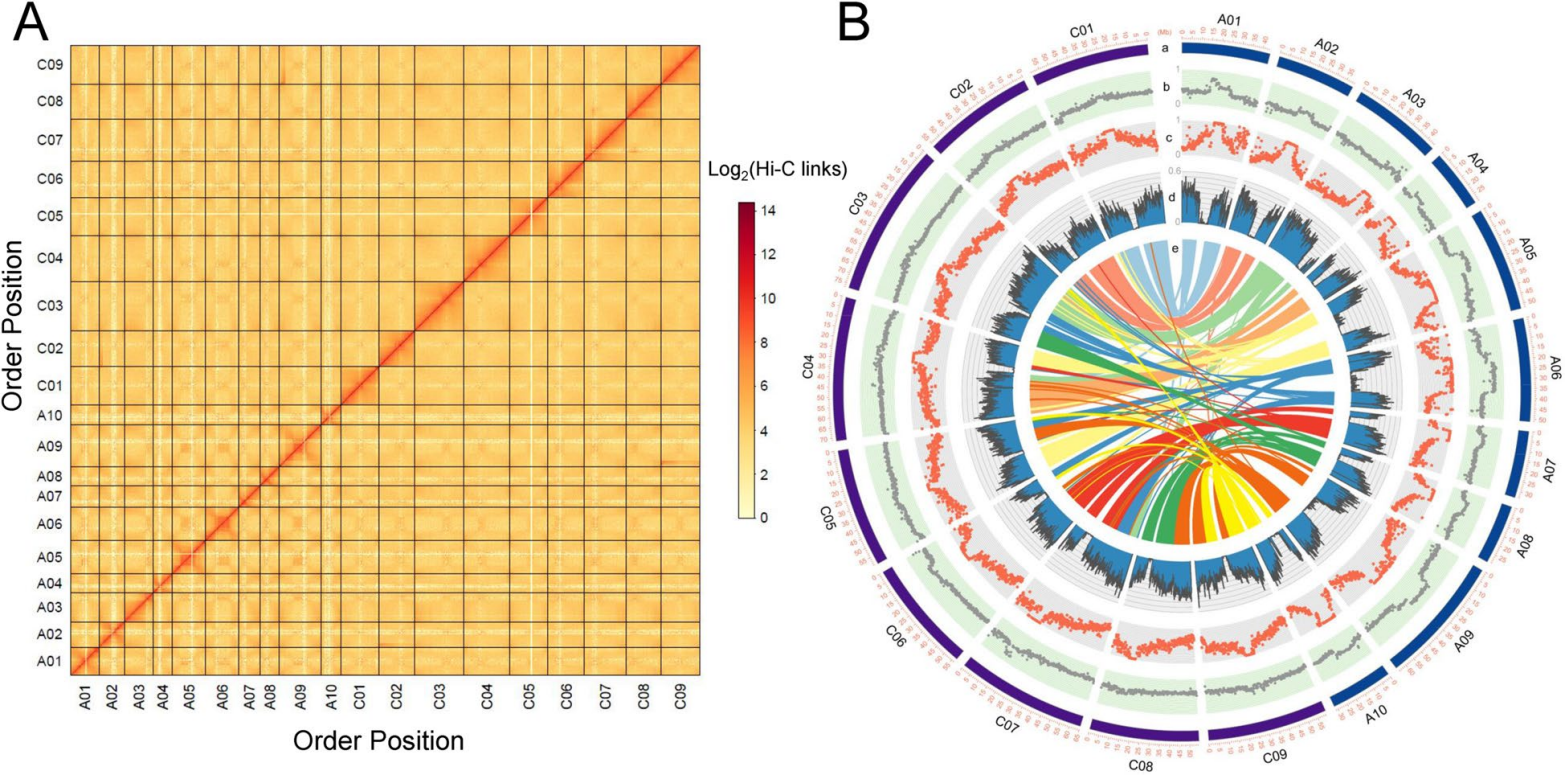
Hi-C assisted assembly of *B. napus* high-GSL variety ZY821 pseudomolecules. **A** Hi-C chromatin interaction heat map of the assembled ZY821 genome. **B** Genomic landscape of ZY821. The tracks from outer to inner show (a) chromosome length, (b) GC content, (c) densities of repetitive sequences, (d) gene density, and (e) syntenic blocks between A_*n*_ and C_*n*_ subgenomes. Window size is 200 kb

We assessed the completeness of the ZY821 assembly using Benchmarking Universal Single-Copy Orthologs (BUSCO) analysis. The assembly contained 99.80% [S:9.7%, D:90.1%, F:0.1%, M:0.1%, n:1614] of the 1,614 conserved Embryophyta genes (Table 1). Meanwhile, the Long Terminal Repeat assembly index (LAI) was 10.91, meeting the standard for reference quality. Consensus quality values (QV) were assessed using Merqury v 2020-01–29 (k-mer = 20) based on Illumina PE150 reads. The QV for the entire assembly (including unanchored sequences) was 33.94, ranging from 43.16 to 62.88 across the 19 chromosomes, with a read mapping rate exceeding 99.65% (Table S3). Finally, alignment of Hi-C reads to the genome produced a contact map that robustly supported the 19 well-assembled chromosomes and revealed no apparent misjoins (Fig. 2A). These evaluations confirm that the ZY821 genome assembly possesses considerable completeness, contiguity, and accuracy.

### Genome annotation

A custom repeat library for the ZY821 genome was constructed using RepeatModeler (v2.0.5), LTR_finder (v1.2), and ltrharvest (v1.6.4). Non-redundant repeats from Repbase and Dfam databases were added to this library. RepeatMasker (v4.1.5) was then used to identify repetitive sequences, annotating a total of 594.71 Mb (58.67%) as repetitive elements (Fig. 2B). Long-terminal repeat retrotransposons (LTR-RTs) constituted the largest class, accounting for 27.67% (280.50 Mb) of the genome, with *Copia* and *Gypsy* elements representing 34.15% and 48.32% of all LTR-RTs, respectively. DNA transposons accounted for 8.58% (86.92 Mb) of the assembly.

Protein-coding gene annotation integrated RNA-seq-based, ab initio, and homology-based predictions. A total of 117,985 protein-coding genes were predicted. BUSCO analysis showed 98.5% completeness for the predicted proteins. Our assembly contains 17,066 and 9,795 more genes than the ZS11 and Darmor-*bzh* v10 assemblies, respectively (Table 2). The average gene length was 2,630 bp, with an average of 6.4 exons per gene.

Functional annotation of the predicted protein-coding genes was performed on four public databases (GO, KEGG, GenBank nr, Uniprot) using DIAMOND. A total of 112,058 (94.98%) genes were annotated in at least one database, and 33,956 genes received annotations in all four databases (Table S4). Leveraging the well-elucidated GSL biosynthesis and metabolism pathways in the closely related model plant *Arabidopsis thaliana*, we identified 588 Brassica orthologs involved in nearly the entire GSL biosynthesis, transport, and regulatory pathway. This identification integrated syntenic analysis (Wang et al. 2012), orthogroup inference (Emms and Kelly 2015), reciprocal best hit (RBH) (Hauser et al. 2016), and phylogenetic analysis (Tamura et al. 2021), followed by manual curation. In summary, the high-quality genome assembly of the elite high-GSL germplasm ZY821 presented here, combined with the previously published genome of the low-GSL representative ZS11, provides a robust foundation for the subsequent analyses in this study.

### Structural rearrangements potentially impacting GSL content

**Fig. 3:**
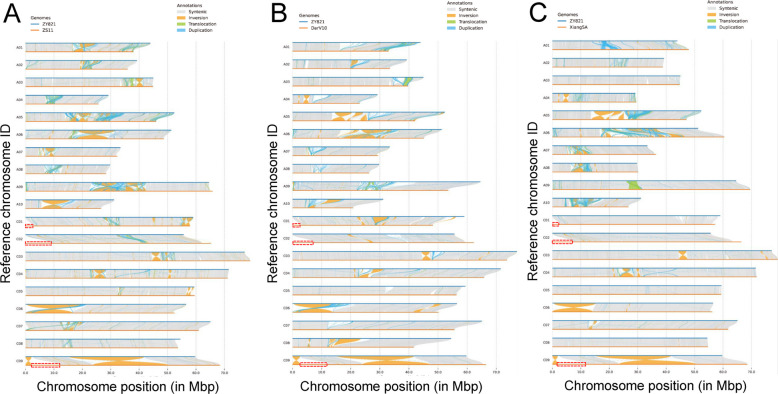
Structural variations between the ZY821 and previous rapeseed genome assemblies. with ZY821 as the reference. **A** ZS11. **B** Darmor-*bzh* V10. **C** Xiang5A

Genomic rearrangements significantly influence agronomic traits and drive phenotypic differentiation (Chalhoub et al. 2014; Zhang et al. 2024). To elucidate the genomic basis underlying the differences between low-GSL (ZS11) and high-GSL (ZY821) varieties, we performed a genome syntenic analysis between ZS11 and ZY821 using NUCmer from the MUMmer package (Marais et al. 2018). While strong collinearity exists between corresponding chromosomes of ZS11 and ZY821, the terminal regions of chromosomes C01, C02, and C09 in ZS11 lack homologous counterparts in the ZY821 assembly (Fig. 3A). This absence is also observed when comparing the ZY821 assembly to other low-GSL varieties, Darmor-*bzh* v10 and Xiang5A (Fig. 3B, C).

To investigate the cause of these missing homologous regions (e.g., genomic deletion or assembly collapse), we mapped high-depth short reads from ZY821 to the ZS11 assembly to detect homoeologous non-reciprocal translocations (HNRTs). HNRT is a type of homoeologous exchange (HE) involving the replacement of a genomic region with its homoeologous counterpart, which can alter gene copy number and expression levels, thereby contributing to phenotypic variation (Chalhoub et al. 2014). As predicted, we identified three large HNRT events in ZY821 where the terminal regions of chromosomes C01, C02, and C09 were replaced by homoeologous segments from chromosomes A01, A02, and A09, respectively (Fig. S4). These findings were corroborated by Hi-C contact maps (Fig. 2A; Fig. S5). Due to their large size (approximately 2 Mb, 9 Mb, and 10 Mb on C01, C02, and C09, respectively), such extensive HE regions often lead to repeat collapse and assembly gaps with current methods. Consequently, we identified candidate genes within the duplicated fragments on A01, A02, and A09 (Table S5). Each gene in these regions potentially has two copies: one physically located on the A_*n*_ subgenome and the other on the corresponding C_*n*_ subgenome due to the HNRT. These three HE regions harbor multiple important genes and QTL that have been previously reported to affect key agronomic traits in rapeseed, including plant architecture, seed number per silique, silique number, disease resistance, drought tolerance and glucosinolate biosynthesis (Raman et al. 2022; Zhang et al. 2024). Among the 588 Brassica GSL-related genes, eight, five, and twelve reside within the HE regions on A01/C01, A02/C02, and A09/C09, respectively. These include genes involved in GSL biosynthesis (e.g., *BnaA01.CYP79B2*, *BnaA02.CYP79A2*, *BnaA01.CYP81F1/3/4*, *BnaA09.SUR1*, *BnaA09.SOT18*), transport (*BnaA09.GTR2a/b*), and regulation (*BnA09.MYB28*, *BnA09.MYB34*). Genes within these HE regions could contribute to the differentiation in GSL content, composition, and distribution between high-GSL (ZY821) and low-GSL (ZS11) varieties, potentially through expression dosage effects of duplicated genes or subfunctionalization between homoeologous gene pairs in ZS11.

Structural variations (SVs) are crucial drivers of plant trait evolution during domestication and breeding. To identify SVs underlying GSL differentiation, we mapped the ZS11 genome to the ZY821 assembly using Minimap2. A total of 31,419 SVs were identified and classified: deletions (16,235), insertions (15,108), inversions (50), and tandem duplications (26) (Table S6). Given that ZY821 possesses an intact GSL metabolism, transport, and regulatory pathway, we hypothesized that key genes might be impaired or deleted in ZS11. We therefore screened for GSL-related genes within deletion regions unique to ZS11. Three genes were identified: *BnaA09.MYB28*, *BnaA08.APR3*, and *BnaC03.ERMO3* (Fig. S6 A-C). Notably, the *Arabidopsis* ortholog of *BnaA09.MYB28* (*MYB28*) is the predominant regulator of aliphatic GSL synthesis, the main component of total GSLs (Sønderby et al. 2007; Gigolashvili et al. 2008). Furthermore, these three genes are also deleted in other low-GSL varieties (Darmor-*bzh* v10 and Xiang5A) (Fig. S6 D, E), suggesting that their deletion is a common feature in low-GSL germplasm. No other known GSL-related genes were found within inversion or duplication fragments.

**Fig. 4:**
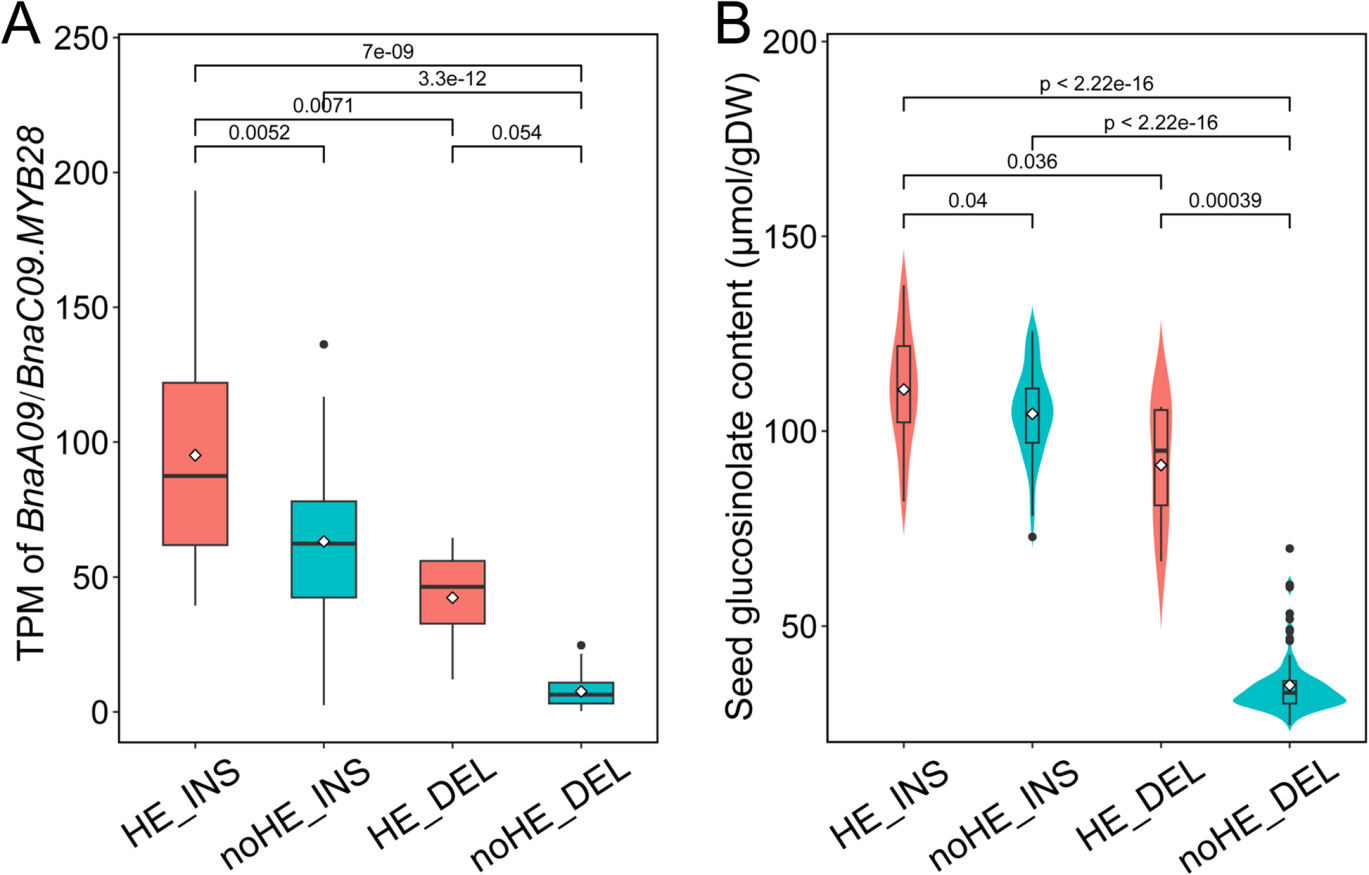
Associations of A09-C09 HEs and SVs at the *BnaA09.MYB28* locus with gene expression (**A**) and seed glucosinolate content (**B**). HE_INS: genotype with A09-C09 HEs occurred and *BnaA09.MYB28* retained; noHE_INS: genotype without A09-C09 HEs occurred and *BnaA09.MYB28* retained; HE_DEL: genotype with A09-C09 HEs occurred and *BnaA09.MYB28* deleted; noHE_DEL: genotype without A09-C09 HEs occurred and *BnaA09.MYB28* deleted

Taken together, *BnaA09.MYB28*, which is involved in both an HNRT and a deletion, may be a strong candidate responsible for the divergence of GSL phenotype on a broad scale in *B. napus*. Thus, we conducted a correlation analysis of genotype, expression level, and GSL contents in a 198-core germplasm panel of *B. napus*. This panel was collected worldwide, exhibited a wide range of GSL content (from 24 to 137 μmol/gDW), and was sequenced to high depth (averaging ~ 40.7-fold coverage) to ensure the reliability of HE identification. Notably, genotypes with either *BnaA09.MYB28* retained on chromosome A09 or those where *BnaC09.MYB28* on chromosome C09 was replaced by *BnaA09.MYB28* via A09-C09 HE showed significantly higher expression of *BnaA09.MYB28* (actually the total of *BnaA09.MYB28* and *BnaC09.MYB28* homoeologs) and higher GSL content in seeds compared to the control in which *BnaA09.MYB28* was deleted and no A09-C09 HE occurred (Fig. 4). Specifically, the genotype that underwent A09-C09 HE but without subsequent *BnaA09.MYB28* deletion on chromosome A09 exhibited the highest *BnaA09.MYB28* transcript abundance and GSL content. This suggests that A09-C09 HE contributed to higher GSL content in *B. napus*, potentially endowing it with stronger environmental adaptability, while the *BnaA09.MYB28* deletion significantly reduced GSL content, improving the economic value of seed products. The combination of these two events endows *B. napus* with a broader range of variation in GSL.

### *BnaA09.MYB28* as a major candidate for GSL content optimization

To further investigate GSL accumulation differences, we performed a comparative analysis of GSL biosynthesis-, transport-, and regulation-related gene expression using a multi-tissue time-course RNA-seq dataset. This dataset comprised 116 samples, spanning roots and leaves at the seedling stage, and roots, stems, and stem leaves at the reproductive stage, and importantly, this dataset included developing seeds and silique walls at 4, 8, 12, 16, 20, 24, 28, 32, 40, 48, and 52 days after pollination (DAP) for both ZY821 and ZS11. This covered nearly the entire rapeseed life cycle, with a focus on seed and silique development to maturation, each with two biological replicates. Expression levels were represented by the average TPM value of replicates. Genes with TPM > 1 were considered expressed.

Among the 588 GSL-related genes, 61 and 31 differentially expressed genes (DEGs) were identified in seedling roots and leaves, respectively (Table S7). At the reproductive stage, 6, 110 (comprising 91, 12, and 17 DEGs in the upper, middle, and lower main stem sections, respectively), and 21 DEGs were found in roots, stems, and stem leaves, respectively. Importantly, during seed GSL accumulation periods, 16, 37, 30, 55, 33, 42, 26, 19, 4, 20, and 11 known GSL-related DEGs were identified at the 11 seed time points (4–52 DAP), respectively. In silique walls, 16, 92, 64, 91, 50, 44, 76, 48, 90, 21, and 11 DEGs were found at the corresponding time points. Furthermore, 133 DEGs were shared between vegetative and reproductive tissues, while 50 and 134 DEGs were specific to vegetative and reproductive tissues, respectively.

**Fig. 5:**
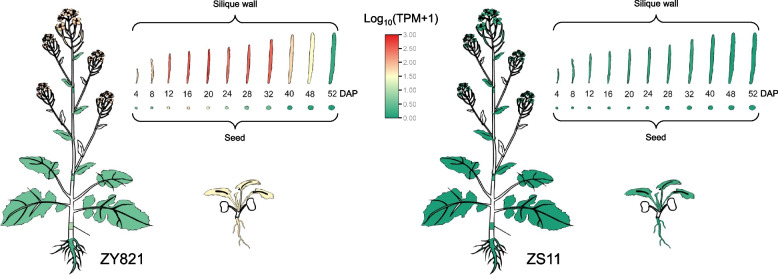
Expression patterns of *BnaA09.MYB28* (including its homoeolog *BnaC09.MYB28*) in low (ZS11) and high (ZY821) GSL varieties. DAP, days after pollination. Color scales represent relative expression levels (log-transformed TPM) from low (green) to high (red)

**Fig. 6:**
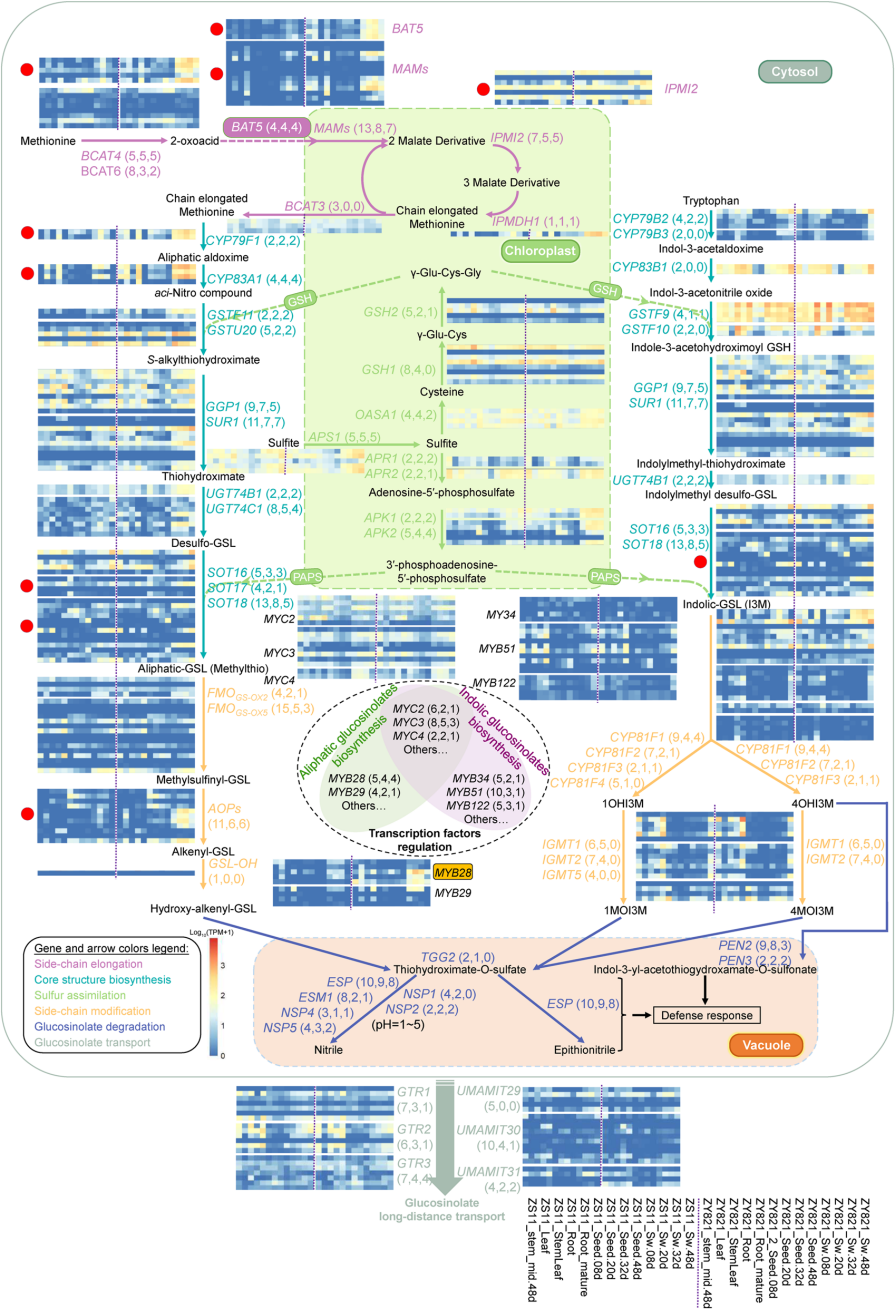
Comparation on gene expression of the whole pathway of GSL biosynthesis and transport in low (ZS11) and high (ZY821) GSL varieties at time-course transcriptome level. Red dots at the left side of heatmap represent genes whose *Arabidopsis* orthologs are reported as *MYB28* targets. The three numbers in brackets in right side of each gene (e.g. Gene (a, b, c) = *MAMs* (13,8,7) are: a = the total number of all homologous genes, b = the number of all homologous genes showed differentially expressed level (DEG), and c = the total number of homologous genes differentially expressed in at least two tissues/stages (VDEG))

To gain a global perspective on GSL differentiation from transcriptomic data, we implemented a stringent criterion to define variety-specific DEGs (VDEGs): genes differentially expressed between ZY821 and ZS11 in at least two corresponding tissues/stages. This identified 17,513 VDEGs, including 226 from the known set of 588 GSL-related genes (Table S7, S8). Among these, 6,839 and 5,177 VDEGs were differentially expressed in at least two stages within developing seeds (SDEGs) and silique walls (SwDEGs), respectively. Notably, 226 VDEGs overlapped with the known GSL-related genes, of which 77 and 120 were SDEGs and SwDEGs, respectively. These SDEG and SwDEG sets likely contribute to seed GSL accumulation divergence. Crucially, the transcription factor *BnaA09.MYB28* (located in the HE region of ZY821 and deleted in ZS11) was both SDEG and SwDEG. Its expression level in ZY821 was substantially higher than its homoeologs across most tissues/stages (Fig. 5; Fig. S7). Moreover, its putative downstream targets in *Arabidopsis* (validated MYB28 targets) — such as *BCAT4* (BnaZ8C050054580, BnaZ8A030039380), *CYP79F1* (BnaZ8A060012150), *CYP83A1* (BnaZ8C040050800, BnaZ8A040009450), and *SOT18* (BnaZ8A070041410) — were also SDEGs and SwDEGs, exhibiting higher abundance in ZY821 (Fig. 6). Other putative downstream GSL-related genes of *BnaA09.MYB28*, like *SOT18* (BnaZ8A090019150), *MAM1* (BnaZ8A020048390), and *CYP83A1* (BnaZ8C040050800, BnaZ8A040009450), also showed significant expression differences in vegetative tissues. Meanwhile, some specific copies of other putative downstream targets of *BnaC02.MYB28* in *B. napus* (a homolog of *BnaA09.MYB28*), like *IPMDH1*, *UGT74B1*, *FMO*_*GS-OX*_*s*, and S*UR1* (Zhou et al. 2023), were also identified as VDEGs. These results collectively suggest that *BnaA09.MYB28* is an underlying core regulator of the GSL metabolic pathway, inducing the expression of multiple GSL synthesis genes to elevate aliphatic GSL content.

### Exploration of other genes potentially impacting GSL content by co-expression network analysis

To identify additional genes potentially influencing GSL content and accumulation patterns, we performed a weighted gene co-expression network analysis (WGCNA) using transcriptome data from all tissues/stages. This identified 93 co-expression modules, each characterized by distinct expression patterns and represented by different colors (Fig. S8A). Modules exhibited varying correlation coefficients with variety, tissue, and developmental stage, while genes within the same module showed correlated expression (Fig. S8B).

**Fig. 7:**
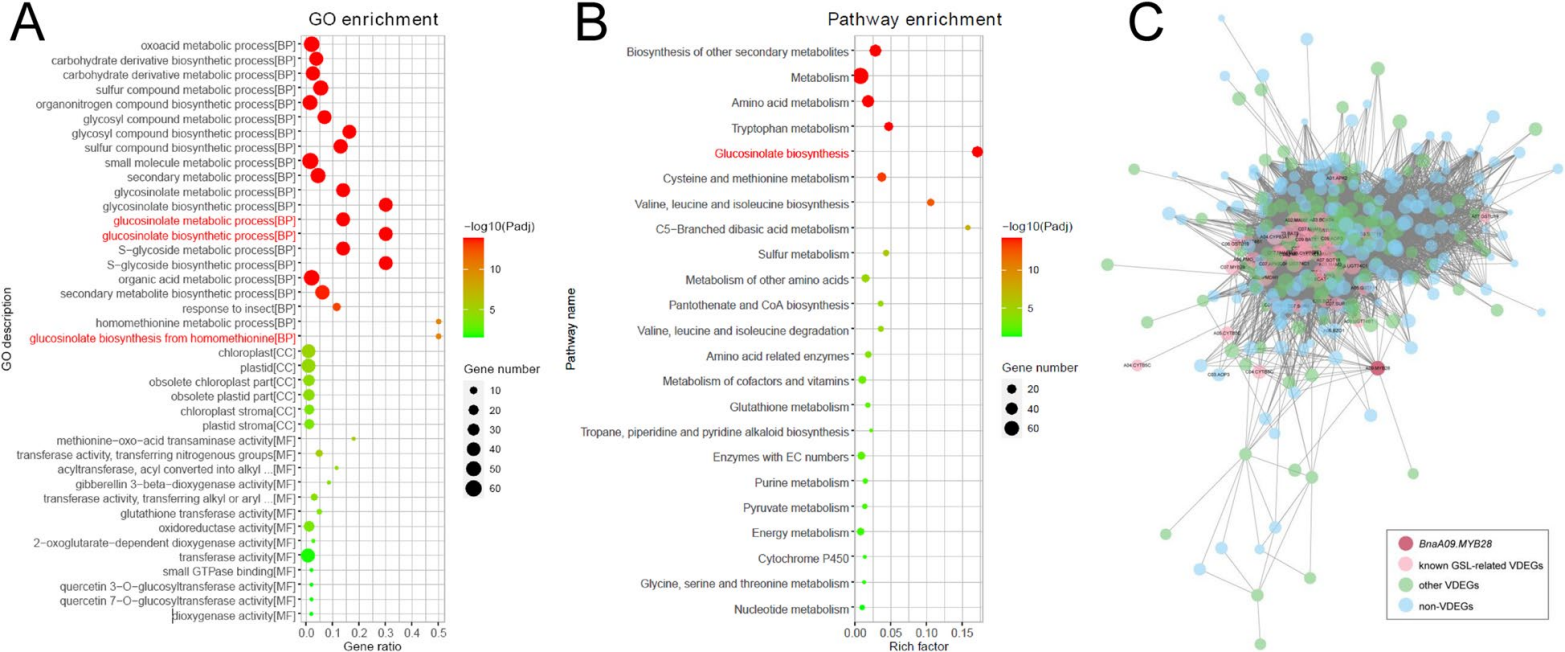
Weighted gene co-expression network analysis identifies important genes potentially impacting on glucosinolate content. **A** GO enrichment analysis of VDEG genes in the Thistle2 module. **B** KEGG pathway analysis of VDEG genes in the Thistle2 module. **C** Weighted gene co-expression network of the Thistle2. VDEGs, differentially expressed genes in transcriptome analysis between ZY821 and ZS11 variety identified repeatedly across multi-tissues or time-courses

Significantly, *BnaA09.MYB28* and 58 other known GSL-related genes were enriched in the Thistle2 module, which contained 488 genes in total, including 248 VDEGs (57 of which are known GSL-related VDEGs). GO enrichment analysis of VDEGs within the Thistle2 module yielded 40 significantly enriched terms after redundancy reduction (21 Biological Process, 6 Cellular Component, 13 Molecular Function). Enriched biological processes were notably related to glucosinolate biosynthetic and metabolic processes (Fig. 7A). KEGG pathway analysis further revealed enrichment for GSL biosynthesis, amino acid metabolism, sulfur metabolism, energy metabolism, and biosynthesis of other secondary metabolites (Fig. 7B). These results indicate that genes within the Thistle2 module likely play important roles in GSL biosynthesis and accumulation, warranting further investigation.

Network visualization revealed a highly interconnected co-expression signature within the Thistle2 module (Fig. 7C). We identified 138 hub genes based on high module membership (|kME|> 0.85), including 42 known GSL-related genes such as *BnaC07.MYB28* and *BnaA09.MYB28*. Other hub genes included transcription factors potentially involved in regulating GSL metabolism (directly or indirectly), such as *BnaA03.LBD15*, *BnaA04.LBD15*, *BnaC04.LBD15*, *BnaA01.MYB85*, *BnaA04.WRKY12*, *BnaC04.WRKY12*, *BnaZ8C040050910* (ERF/AP2 family), *BnaC07.VAL2*, and *BnaC08.IAA5*. Additionally, hub genes encoding enzymes like BnaZ8A020013640 (aspartate kinase 3), BnaZ8A030028630 (methionine sulfoxide reductase B3), BnaZ8C090053670 (sulfotransferase 12), and BnaZ8C090075400 (sulfate transporter 2;1) may influence substrate biosynthesis, utilization, or secondary modifications in GSL pathways. Furthermore, we screened 197 genes within the Thistle2 module showing high correlation (weight > 0.1) with *BnaA09.MYB*28 (Table S9). Besides the 22 known GSL-related genes (37 total in the 197) whose *Arabidopsis* orthologs are reported as *MYB28* targets, other genes such as *BnaAPK2s*, *BnaCYTB5Cs*, *BnaGSTU19s*, *BnaFMO*_*GS-OX1s*_, *BnaC07.*SUR1, *BnaA02.IPMDH1*, *BnaA05.GSTF11*, *BnaA05.IPMI2*, and *BnaA05.BZO1*, along with previously unreported GSL-related genes in this dataset, may also have their transcript abundance induced by *BnaA09.MYB28* to participate in GSL metabolism*.*

## Discussion

Glucosinolates (GSLs) are characteristic secondary metabolites of Brassicales plants, serving crucial defense functions against fungal pathogens and herbivorous pests. However, their high accumulation in seeds diminishes the quality of Brassica crops, particularly rapeseed. The ideal GSL distribution profile for oilseed crops like rapeseed entails low levels in seeds and high levels in green organs. Despite dedicated efforts by researchers and breeders over the past two decades, achieving this goal has proven challenging (Tu et al. 2025; Zhang et al. 2024). This stems from the scarcity of natural germplasm exhibiting the desired profile and an incomplete understanding of the genetic basis underlying GSL differences between high- and low-GSL varieties. A significant factor hindering this understanding has been the lack of high-quality reference genomes representing high-GSL germplasm, which is essential for comprehensive genome exploration and comparative analysis, despite the availability of assemblies for low-GSL varieties like ZS11, Darmor-*bzh*, and Xiang5A.

To address this gap, we generated a chromosome-level genome assembly for ZY821, an elite high-GSL variety, by integrating PacBio HiFi sequencing with Hi-C scaffolding. The resulting assembly spans over 1,013 Mb, with a contig N50 of 14.23 Mb and 99.80% BUSCO completeness. This represents a significant improvement in contiguity, completeness, and accuracy compared to previous assemblies like Darmor-*bzh* v4.1 and v10, and approaches the quality of the gap-free Xiang5A assembly. We annotated 594.71 Mb (58.67%) of repetitive sequences and predicted 117,985 protein-coding genes. This high-quality genomic resource provides a valuable foundation for genomic studies in *B. napus*. Furthermore, we comprehensively identified genes involved in the GSL pathway within the ZY821 genome, establishing a basis for subsequent comparative genomic and for transcriptomic analyses between high- and low-GSL varieties and future functional gene studies.

Chromosomal structural rearrangements, including SVs, play a pivotal role in trait variation and environmental adaptation. To elucidate the genomic basis of GSL differentiation between two representative varieties, we performed comparative genomic analyses using ZY821 (high-GSL) and ZS11 (low-GSL). Our results revealed three large homoeologous non-reciprocal translocation (HNRT)-type homoeologous exchange (HE) events on chromosomes C01, C02, and C09 in the ZY821 genome, alongside numerous SVs between the two varieties. GSL-related genes located within these HE or SV regions were identified as potential contributors to GSL content divergence, as their function or expression may be altered. For example, in ZY821, the *BnaC09.MYB28* locus was replaced by *BnaA09.MYB28* due to an HE event on chromosome C09 (where an A09 fragment replaced its homoeologous C09 counterpart). Among the three genes deleted in ZS11, *BnaA09.MYB28* is particularly significant. Its *Arabidopsis* ortholog (*MYB28*) encodes an R2R3-MYB transcription factor that acts as the master regulator of aliphatic GSL biosynthesis (Sønderby et al. 2007; Gigolashvili et al. 2008). The conserved deletion of *BnaA09.MYB28* in other low-GSL varieties like Darmor-*bzh* v10 and Xiang5A suggests a critical and conserved role in GSL biosynthesis and likely underpins the phenotypic differences. Our genotyping of HEs and SVs at the *BnaA09.MYB28* locus in the natural population supports this *BnaA09.MYB28* (including its homoeolog *BnaC09.MYB28*) expression is upregulated when the HE is present, but downregulated when the *BnaA09.MYB28* deletion allele is present, leading to variation in GSL accumulation.

Subsequent multi-tissue time-course comparative transcriptome analysis strongly supported this finding and illuminated the underlying mechanism. During seed development, *BnaA09.MYB28* was identified as both a seed DEG (SDEG) and silique wall DEG (SwDEG). In ZY821, it exhibited high expression levels in silique walls, gradually increasing after pollination, peaking at 20 days after pollination (DAP), and then declining. Crucially, its putative downstream target genes — such as *BnaC05.BCAT4*, *BnaA03.BCAT4*, *BnaA06.CYP79F1*, *BnaC04.CYP83A1*, *BnaA04.CYP83A1*, and *BnaA07.SOT18* (whose *Arabidopsis* orthologs are validated MYB28 targets) — were also SDEGs and SwDEGs. These targets displayed similar expression patterns and significantly higher abundance in ZY821 compared to ZS11.

Given the structural differences between the ZY821 and ZS11 genomes, mapping ZS11 RNA-seq reads to the ZY821 reference genome may introduce reference bias, particularly for genes in HE regions. This approach could potentially influence expression estimates or inflate the number of DEGs in regions affected by structural rearrangements. However, for the key gene *BnaA09.MYB28*, together with two other GLS-related genes in SV regions, their complete absence in ZS11 was independently confirmed by whole-genome resequencing, ensuring that the observed expression difference is genuine and not an artifact of mapping bias. Furthermore, the differential expression patterns of its putative downstream targets were consistent with expectations based on the presence or absence of *BnaA09.MYB28* regulation. Furthermore, among the 588 GSL-related genes (including 226 VDEGs) identified in the ZY821 genome, only 25 (including 13 VDEGs) located in HE regions, just accounting for 4.25% (5.75%). Taken together, while reference bias may affect expression estimates for a small subset of GLS-related genes in genomic variant regions, the overall conclusions of this study, particularly the central role of *BnaA09.MYB28* in regulating GSL content, remain robust. Although our results confirmed that *BnaA09.MYB28* is a major positive regulator of GSL content in *B. napus* and that its presence or absence is a primary determinant of the high- versus low-GSL phenotype. Nevertheless, further validation through targeted editing of *BnaA09.MYB28* in ZY821 and assays like Y1H, EMSA, or dual-luciferase assays is warranted to confirm its direct activation of downstream genes within the GSL biosynthesis pathway.

Our findings indicate that the deletion of *BnaA09.MYB28*, caused by an SV event, leads to reduced expression of numerous downstream GSL-biosynthesis genes and consequently low GSL synthesis. This provides a genetic explanation for the scarcity of natural germplasm exhibiting the ideal phenotype of high GSLs in green tissues and low GSLs in seeds. As a core TF regulating aliphatic GSL accumulation, fine-tuning the expression level of *BnaA09.MYB28*, modifying its tissue-specific accumulation pattern, or disrupting its interactions with specific downstream biosynthesis genes through precise genome design could optimize GSL distribution in *B. napus*. While the SV leading to *BnaA09.MYB28* deletion has been pervasively utilized during double-low breeding, albeit unconsciously, the specific goal of increasing GSL content in green tissues by leveraging *BnaA09.MYB28* (for example, by combining the A09-C09HE with mutations in GSL seed-loading transporters like *BnaGTRs* and/or *BnaUMAMITs* to further enhances GSL biosynthesis in source tissues and uncouple with seed loading) has not yet been realized, representing a significant untapped breeding potential.

The multi-tissue time-course comparative transcriptome analysis also revealed enrichment of numerous known GSL-related genes within the differentially expressed gene (DEG) sets. This not only validates the reliability of the RNA-seq data and analytical methods, but also underscores the critical roles of these DEGs in GSL biosynthesis and metabolism. The extensive sampling across tissues and developmental stages in both ZY821 and ZS11 enabled the identification of DEGs recurring across multiple samples. This allowed us to apply a stringent criterion to define variety-specific DEGs (VDEGs), which are strong candidates underlying trait variation, including GSL content, between ZY821 and ZS11. This VDEG set, particularly the SwDEGs differentially expressed in silique walls during seed development, significantly narrows the scope for identifying novel GSL-related genes and further emphasizes the importance of known GSL pathway genes.

To uncover additional genes potentially influencing GSL content, we performed weighted gene co-expression network analysis (WGCNA). The Thistle2 module, enriched with known GSL-related genes including *BnaA09.MYB28*, was analyzed in detail. Subsequent GO and KEGG pathway enrichment analyses of VDEGs within this Thistle2 module confirmed its significant association with GSL biosynthetic and metabolic processes. Consequently, the hub genes (showing the highest connectivity) and VDEGs highly correlated with *BnaA09.MYB*28 within this module represent prime candidates for involvement in GSL biosynthesis and metabolism, warranting detailed functional investigation.

## Conclusion

This study presents a high-quality genome assembly for the high-GSL representative variety ZY821, providing a robust resource for future gene functional studies and crop improvement, particularly for GSL trait optimization. Leveraging this genome alongside the previously published genome of the elite low-GSL variety ZS11, we employed comparative genomic analyses to identify genes potentially underpinning GSL differentiation. We established *BnaA09.MYB*28 as an underlying major contributor, as its deletion via an SV on chromosome A09 in ZS11 leads to reduced expression of numerous downstream genes, including orthologs of nearly all known targets of *Arabidopsis MYB28*. This key finding was collaboratively supported by multi-tissue time-course comparative transcriptome analysis. Furthermore, the A09-C09 HE containing *BnaA09.MYB*28 further amplified the differences of glucosinolate accumulation with the population. Additionally, this research identified novel putative targets of *BnaA09.MYB*28 and potential new genes involved in the GSL pathway. Collectively, this study provides valuable genomic data and novel insights into the genetic regulation of GSL accumulation in *B. napus*.

## Materials and methods

### Plant materials and sample preparation

The *B. napus* varieties in this study were planted in Yangluo (Wuhan city, 30°43'N and 114°31'E) in the growth seasons. After at least six generations of purification by self-pollination, the ZY821 and ZS11 varieties were then used for this study. Crop management of field experiments followed the standard protocol of China National Rapeseed Variety Field Test. Samples from specific tissues or periods mentioned in this study were collected in liquid nitrogen and stored at − 80 ℃ in a refrigerator until use.

### DNA extraction and DNA sequencing

For PacBio long-read sequencing, young leaves of ZY821 variety were harvested from three-week-old seedlings and immediately flash frozen in liquid nitrogen. High molecular weight DNA was isolated using the Blood & Cell Culture DNA Midi Kit (QIAGEN). DNA fragments of 10–20 kb in length were selected by BluePippin electrophoresis (Sage Sciences) and purified by AMPure PB magnetic beads. The High-fidelity (HiFi) libraries were constructed using SMRTbell Express Template Prep Kit 2.0 and sequenced on PacBio Sequel IIe platform (Pacific Biosciences, Menlo Park, USA) by Beijing Novogene Company (Beijing, China). At least 30.35 Gb HiFi reads with N50 sizes of 17,588 bp were obtained using the CCS (Circular Consensus Sequencing) software with default parameters (https://ccs.how/).

For whole genome re-sequencing of ZY821 variety, total DNA was extracted from three-week-old seedlings by the cetyltrimethylammonium bromide (CTAB, Sigma Aldrich) method. Quality, quantity and molecular size of DNA samples were assessed using Nanodrop (Thermofisher). A paired-end sequencing (PE150) library was generated with an insert size of approximately 350 bp following the manufacturer’s standard protocol (Illumina) and sequenced on the Illumina HiSeq platform by Beijing Novogene Company (Beijing, China).

For Hi-C sequencing, 5 g of fresh leaves collected from three-week-old seedlings were used for library construction. The DNA crosslinking was performed by 1% formaldehyde. The linked DNA was digested with *Dpn*II restriction endonuclease (NEB, Cat#0543), and the DNA ends were marked by biotin-14-dCTP and ligated by T4 DNA Ligase. The ligated DNA was sheared into 300–600 fragments by sonication and sequenced on the Illumina HiSeq system (PE 150). About 90.37 Gb raw data were obtained for ZY821.

### Plant tissue collection, RNA extraction and comparative analysis for RNA-Seq

The RNA-Seq samples from 29 tissues/stages in both high GSL variety ZY821 and low GSL variety ZS11 were collected and sequenced. In brief, tissues from seeds and silique walls at 4, 8, 12, 16, 20, 24, 28, 32, 40, 48 and 52 days after pollination (DAP) on main inflorescence, together with samples from root and leaf at seeding stage and root, stem (three samples from the upper, middle and lower parts of the main stem) and stemleaf at reproductive stage, were collected in the two represented varieties respectively and immediately frozen by liquid nitrogen. Two biological replicates per sample and each replicate obtained from four independent plants, were pooled for transcriptome sequencing. Total RNA was extracted using the RNeasy Plant Mini Kit (QIAGEN) according to the manufacturer’s instructions. Library of mRNA were prepared and sequenced using HiSeq 150-bp paired-end Illumina RNA-Seq protocol. The low-quality RNA-seq reads were firstly removed by Trimmomatic software (v0.36) (Bolger et al. 2014). Then, the clean data were mapped to ZY821 genome using Hisat2 (v2.1.2) with default parameters (Kim et al. 2015). Gene expression level (TPM) was calculated using StringTie software (v1.3.5) with default settings (Pertea et al. 2015). Further, DEGs were identified between ZY821 and ZS11 for each tissue/time point using ballgown (|log2foldchange|≥ 1 and adjusted *P*-value (padj) < 0.05).

### Genome assembly

To generate ZY821 genome assembly, the PacBio HiFi reads were de novo assembled by using hifiasm (v0.16.121) (Cheng et al. 2021) with default parameters and the Hi-C data was used to anchor the genome sequence through ALLHiC (Zhang et al. 2018). Then the clean Hi-C reads were mapped to the genome assembly using Juicer (v1.9.922) (Durand et al. 2016b). The unique mapped reads were taken as input for 3D-DNA pipeline (v18011423) (Dudchenko et al. 2017) and some manual adjustments were made by using JuicerBox (v1.11.0824) (Durand et al. 2016a). Finally, a total of 19 pseudo-chromosomes was obtained, which contained 96.53% of the assembled contigs for ZY821.

### Assembly validation and quality assessment

To evaluate the accuracy of ZY821 assembly, Illumina reads were mapped to the assembly with BWA v0.7.17 (Li and Durbin 2009) and the mapping rate was calculated using SAMtools v1.18 (Li et al. 2009). The LTR Assembly Index (LAI) calculated from LTR_retriever v2.9.4 (Ou and Jiang 2018) was used for the evaluation of the genome assembly quality. BUSCO v5.5.0 (Manni et al. 2021) was introduced to assess the assembly completeness with the embryophyta_odb 10 lineage database. Merqury v1.3 (Rhie et al. 2020) was applied to assess the base-level accuracy and completeness.

### Genome annotation

For repeat annotation, homology alignment and de novo search techniques were integrated to comprehensively identify whole-genome repeats. Custom repeat libraries were constructed by screening the ZY821 genome using RepeatModeler-2.0.2a (Flynn, 2020), LTR_finder (Zhao and Hao 2007) and ltrharvest (Ellinghaus et al. 2008) with the default parameters. Then, the non-redundant repeats from Repbase (Jurka et al. 2005) and Dfam (Wheeler et al. 2013) databases were extracted and added to the custom libraries. Then, this refined custom library was then furnished to RepeatMasker for comprehensive repeat annotation (Tarailo‐Graovac and Chen 2009).

The gene structures of the soft-masked ZY821 genome were annotated through a combination of three approaches (ab initio predictions, homologue proteins and transcriptome data) using MAKER (v2.31.10) (Cantarel et al. 2008). Ab initio gene prediction based on the soft-masked genome was first performed with Augustus (v3.3.2) (Hoff and Stanke 2019), SNAP (v 2006-07–28) (Korf 2004) and GlimmerHMM (v3.0.4) (Majoros et al. 2004). Proteins from *B. rapa* (Chiifu-401–42, v3.0), *B. oleracea* (To1000, v2.0), *B. napus* (ZS11.v1 and Darmor-*bzh*.v5) and *A. thaliana* (TAIR10) were used for the homologue protein-based annotation of the ZY821 genome by Exonerate (v2.2.0) (Slater and Birney 2005). The RNA-seq evidence, ab initio prediction and homolog evidence were fed to MAKER to generate the final gene set.

Functional annotation of protein-coding genes was conducted based on four public databases, including GO (http://geneontology.org/), KEGG (https://www.kegg.jp/), GenBank nr (https://www.ncbi.nlm.nih.gov/) and Uniprot (https://www.uniprot.org/), using DIAMOND (v2.0.13.15147) (Buchfink et al., 2021).

### Annotation of GSL-related genes in the ZY821 genome

To systematically identify GSL-related genes in the ZY821 genome, we employed an integrated orthology inference strategy combining syntenic analysis, reciprocal best hits (RBH), orthogroup inference, and phylogenetic analysis, followed by manual curation. A set of 109 experimentally validated GSL genes from *Arabidopsis*, together with annotated GSL-related genes from Darmor-bzh.v5 and ZS11.v1, were used as queries. Syntenic analysis was performed using MCScan (Python version) with default parameters. RBH analysis was conducted using mmseqs easy-rbh with default parameters. OrthoFinder (v2.5.5) was used to cluster genes into orthogroups between *Arabidopsis* and ZY821. The Orthogroups with two or more *Arabidopsis* genes were subjected to phylogenetic analysis. For a small subset of genes with inconsistent results across methods, Neighbor-Joining trees were constructed using MEGA11 with 1,000 bootstrap replicates; genes clustering with known *Arabidopsis* GSL genes (bootstrap ≥ 70%) were prioritized. Following integration, deduplication, and manual curation, a final set of 588 GSL-related genes was established in ZY821.

### Structural variation identification

To identify SVs, other previous released high-quality rapeseed genomes were mapped to our assembled ZY821 genome by using Minimap2 (v2.26-r1175) (Li 2018) with the parameter -ax asm5. Then SVIM-asm (v1.0.3) (Heller and Vingron 2021) was adopted to call SVs based on the resulting alignment.

### GO and KEGG enrichment analyses

The functional annotation of protein sequences used for the GO and KEGG enrichment analyses in ZY821 was based on the eggNOG-mapper (v2.0.1) program. GO enrichment and KEGG pathway analysis were performed using TBtools and visualized with ggplot2.

### Gene co-expression network analysis

Weighted gene co-expression network analysis was performed with the WGCNA package in R (Langfelder and Horvath 2008). Weighted networks with scale-free topology were constructed using automatic network construction function (blockwiseModules) with default parameters, apart from the soft threshold power of 8, TOMtype was unsigned, mergeCutHeight was 0.25 and minModuleSize was 30. Obtained modules were color-coded and selected modules were visualized by Cytoscape software (Shannon 2003).

### Glucosinolate extraction and quantification

Sample from leaf and root at seeding stage and cauline leaf, stem, silique wall and seed at reproductive stage were harvested in ZY821 and ZS11 variety. Each biological replicate combined samples from six plants and each tissue contained at least three biological replicates. Sample of each biological replicate was placed into a 50-mL tube with 25 mL of 90% methanol and fifteen 3-mm ball bearings. After ground into fine powder in a paint shaker by high-speed agitation and centrifuged, the supernatant was transferred to a 15-mL column that contained 1 mL aqueous solution of DEAE Sephadex A-25 (Sigma-Aldrich). The Sephadex-bound glucosinolate were eluted by incubation with Sulfatase (Sigma-Aldrich). The GSL content was quantified by high-performance liquid chromatography (HPLC, Agilent) using 2-propenyl GSL (Sinigrin, Sigma-Aldrich) as an internal standard according to previously described method (Kliebenstein et al. 2001). The population seed GSL data were derived from our previously published study (Zhang et al. 2024).

## Supplementary Information

Supplementary Material 1: Figure S1. Distribution of telomeres and gaps in ZY821 genome. Figure S2. Synteny between ZY821 assembly and other published genome assemblies. Figure S3. Synteny between Xiang5A assembly and other published genome assemblies. Figure S4. Identification of HNRT-HE based on read-depth approach on whole genome resequencing data. Figure S5. Heatmap showing Hi-C interactions under a resolution of 200-kb. Figure S6. Identification and characterization of GSL-associated genes lost in ZS11. Figure S7. Expression patterns of all BnaMYB28 family members in low (ZS11) and high (ZY821) GSL varieties with ZY821 as reference. Figure S8. Weighted gene co-expression network analysis identifies genes potentially functioning in GSL metabolic pathways.

Supplementary Material 2: Table S1. Information of predicted telomeres and gaps in ZY821. Table S2. Chromosome lengths (bp) for each chromosome of ZY821 and other reference genome. Table S3. Consensus quality values (QV) assessed by Merqury (k-mer=20) using Illumina PE150 reads. Table S4. Statistics of predicted protein-coding genes. Table S5. Genes located in the HE regions of A01-C01, A02-C02 and A09-C09 in ZY821. Table S6. Statistics of structural variants (SVs) between ZY821 and ZS11 genomes. Table S7. Summary of glucosinolate-related differentially expressed genes across 29 tissues in ZY821 and ZS11. Table S8. Summary statistics of differentially expressed genes across 29 tissues in ZY821 and ZS11. Table S9. Candidate genes related to BnaA09.MYB28 identified in the Thistle2 module.

## Data Availability

The supporting data for all Figures and Tables are available in Supplemental Figs. 1–8 and Supplemental Tables 1–9. All genome assembly, annotations, SV information and RNAseq data are available at the BnaOmics Portal (https://BnaOmics.ocri-genomics.net). The material will be available from the corresponding author upon reasonable request.

## Abbreviations

BUSCO: Benchmarking Universal Single-Copy Orthologs
CTAB: Cetyltrimethylammonium bromide
DAP: Days after pollination
DEG: Differentially expressed gene
GSL: Glucosinolate
HE: Homoeologous exchange
Hi-C: High-throughput chromosome conformation capture
HNRT: Homoeologous non-reciprocal translocation
HPLC: High-performance liquid chromatography
LAI: Long Terminal Repeat assembly index
SV: Structural variant
TF: Transcription factor
TPM: Transcripts per million
VDEG: Variety-specific differentially expressed gene
WGCNA: Weighted gene co-expression network analysis
