# Supplementary material for: Comparative genomics and time-course transcriptomics uncover homoeologous exchange events reshaping glucosinolate metabolism in Brassica napus
Source: Mol Hortic. 2026 Aug 4;6:52. doi: 10.1186/s43897-026-00241-y (PMC13435445; doi:10.1186/s43897-026-00241-y)
Supplement: Supplementary file 1 [file 43897_2026_241_MOESM1_ESM.docx]

**Title Page**

**Comparative genomics and time-course transcriptomics uncover homoeologous exchange events reshaping glucosinolate metabolism in *Brassica napus***

Yizhou He^1,2†^, Zetao Bai^1†^, Zengfeng Wang^1,3†^, Yu-Xuan Bai^4^, Qinlin Ke^1^, Fan Liu^1^, Chaobo Tong^1^, Aixia Gu^3^, Marta Francisco^5^, Deyang Xu^6^, Lijiang Liu^1,^*, Qing-Yong Yang^2,^*, Shengyi Liu^1,^*, Wen-Hui Lin^4,7^*, Yuanyuan Zhang^1,^*

^1^The Key Laboratory of Biology and Genetic Improvement of Oil Crops, The Ministry of Agriculture and Rural Affairs of PRC, Oil Crops Research Institute, Chinese Academy of Agricultural Sciences, Wuhan 430062, Hubei, China

^2^National Key Laboratory of Crop Genetic Improvement, Hubei Hongshan Laboratory, Huazhong Agricultural University, Wuhan 430070, Hubei, China

^3^College of Horticulture, State Key Laboratory of North China Crop Improvement and Regulation, Key Laboratory of Vegetable Germplasm Innovation and Utilization of Hebei, Hebei Agricultural University, Baoding 071001, Hebei, China

^4^State Key Laboratory of Microbial Metabolism, Joint International Research Laboratory of Metabolic and Developmental Sciences, School of Life Sciences and Biotechnology, Shanghai Jiao Tong University, Shanghai 200240, China

^5^Group of Genetics, Breeding and Biochemistry of Brassicas, Misión Biológica de Galicia, Pontevedra 36080, Spain

^6^DynaMo Center, Department of Plant and Environmental Sciences, Faculty of Science, University of Copenhagen, Frederiksberg, Denmark.

^7^Key Laboratory of Western Resources and Modern Biotechnology of the Ministry of Education, Key Laboratory of Biotechnology of Shaanxi Province, College of Life Sciences, Northwest University, Xi’an 710069, Shaanxi, China

*Corresponding author: Yuanyuan Zhang (zhangyy@caas.cn), Wen-Hui Lin ([whlin@sjtu.edu.cn](mailto:whlin@sjtu.edu.cn)), Shengyi Liu ([liusy@oilcrops.cn](mailto:liusy@oilcrops.cn)), Qing-Yong Yang ([yqy@mail.hzau.edu.cn](mailto:yqy@mail.hzau.edu.cn)) and Lijiang Liu ([liulijiang@caas.cn)](mailto:liulijiang@caas.cn)).

^†^These authors contributed equally to this work.

**Supplemental Figures**

**Figure S1.** Distribution of telomeres and gaps in ZY821 genome.

**Figure S2.** Synteny between ZY821 assembly and other published genome assemblies.

**Figure S3.** Synteny between Xiang5A assembly and other published genome assemblies.

**Figure S4.** Identification of HNRT-HE based on read-depth approach on whole genome resequencing data with ZS11 as the reference.

**Figure S5.** Heatmap showing Hi-C interactions under a resolution of 200**-**kb.

**Figure S6.** Identification and characterization of GSL-associated genes lost in ZS11.

**Figure S7.** Expression patterns of all *BnaMYB28* family members in low (ZS11) and high (ZY821) GSL varieties with ZY821 as reference.

**Figure S8.** Weighted gene co-expression network analysis identifies genes potentially functioning in GSL metabolic pathways.

**Figure S1. Distribution of telomeres and gaps in ZY821 genome.** Blue triangles and orange squares represent telomeres and gaps respectively.

**Figure S2. Synteny between ZY821 assembly and other published genome assemblies.** The pseudo-chromosomes of other assemblies were aligned to the ZY821 assembly by minimap2 with default parameters.

**Figure S3. Synteny between Xiang5A assembly and other published genome assemblies.** The pseudo-chromosomes of other assemblies were aligned to the Xiang5A assembly.

**Figure S4. Identification of HNRT-HE based on read-depth approach on whole genome resequencing data with ZS11 as the reference**. Bin width: 10-kb.

**Figure S5. Heatmap showing Hi-C interactions under a resolution of 200-kb.** **A.** Interactions of the chromosomes A01 and C01. **B.** Interactions of the chromosomes A02 and C02. **C.** Interactions of the chromosomes A09 and C09. Red lines below the heatmaps highlighting the regions in A*_n_* subgenome which replaced their homoeologous counterparts in C*_n_* subgenome.

**Figure S6.** **Identification and characterization of GSL-associated genes lost in ZS11. A-C.** genome local collinearity around *BnaA09.MYB28* between ZY821 and previous rapeseed genome assemblies ZS11 (**A**), X5A (**B**) and Darmor v10 (**C**). **D-E.** local collinearity between ZY821 and ZS11 around *BnaA08.APR3* (**D**) and *BnaC03.ERMO3* (**E**).

**Figure S7. Expression patterns of all *BnaMYB28* family members in low (ZS11) and high (ZY821) GSL accessions, with ZY821 as reference.** Sw, silique wall; DAP, days after pollination.

**Figure S8. Weighted gene co-expression network analysis identifies genes potentially functioning in GSL metabolic pathways. A.** Dendrogram showing co-expression modules (clusters) identified across multi- tissues/-stages in low GSL (ZS11) and high GSL (ZY821) varieties. **B.** Expression heatmaps of genes clustered in Thistle2 module.
